# Negative regulation of EB1 turnover at microtubule plus ends by interaction with microtubule-associated protein ATIP3

**DOI:** 10.18632/oncotarget.6196

**Published:** 2015-10-20

**Authors:** Lauriane Velot, Angie Molina, Sylvie Rodrigues-Ferreira, Anne Nehlig, Benjamin Pierre Bouchet, Marina Morel, Ludovic Leconte, Laurence Serre, Isabelle Arnal, Diane Braguer, Ariel Savina, Stéphane Honore, Clara Nahmias

**Affiliations:** ^1^ Inserm U981, Institut Gustave Roussy Department of Molecular Medicine, Villejuif, France; ^2^ Université Paris-Saclay, Villejuif, France; ^3^ CNRS UMR8104, Institut Cochin, Paris, France; ^4^ Cell Biology, Faculty of Science, Utrecht University, Padualaan, CH Utrecht, The Netherlands; ^5^ Cell and Tissue Imaging Core Facilty, PICT-IBiSA, CNRS UMR144 Institut Curie, Centre de Recherche, Paris, France; ^6^ Inserm U836, Grenoble Institut des Neurosciences, Grenoble, France; ^7^ Aix Marseille Université, Inserm, CRO2 UMR_S 911, Marseille, France; ^8^ APHM, Hôpital Timone, Marseille, France; ^9^ Scientific Partnerships Roche SAS, Boulogne Billancourt, France

**Keywords:** EB1, MTUS1, protein interaction, +TIP, microtubule dynamics

## Abstract

The regulation of microtubule dynamics is critical to ensure essential cell functions. End binding protein 1 (EB1) is a master regulator of microtubule dynamics that autonomously binds an extended GTP/GDP-Pi structure at growing microtubule ends and recruits regulatory proteins at this location. However, negative regulation of EB1 association with growing microtubule ends remains poorly understood. We show here that microtubule-associated tumor suppressor ATIP3 interacts with EB1 through direct binding of a non-canonical proline-rich motif. Results indicate that ATIP3 does not localize at growing microtubule ends and that *in situ* ATIP3-EB1 molecular complexes are mostly detected in the cytosol. We present evidence that a minimal EB1-interacting sequence of ATIP3 is both necessary and sufficient to prevent EB1 accumulation at growing microtubule ends in living cells and that EB1-interaction is involved in reducing cell polarity. By fluorescence recovery of EB1-GFP after photobleaching, we show that ATIP3 silencing accelerates EB1 turnover at microtubule ends with no modification of EB1 diffusion in the cytosol. We propose a novel mechanism by which ATIP3-EB1 interaction indirectly reduces the kinetics of EB1 exchange on its recognition site, thereby accounting for negative regulation of microtubule dynamic instability. Our findings provide a unique example of decreased EB1 turnover at growing microtubule ends by cytosolic interaction with a tumor suppressor.

## INTRODUCTION

Microtubules (MTs) are polarized structures that continuously switch between periods of polymerization and depolymerization at their growing (plus) ends. This process, termed MT dynamic instability, allows rapid reorganization of the MT cytoskeleton during essential cell functions such as cell polarity and migration, mitosis and intracellular transport of proteins and organelles. Alterations in MT dynamic instability parameters lead to defects in MT targeting, mitotic spindle formation and chromosome segregation, with subsequent consequences on cancer initiation and progression.

MT dynamic instability is tightly regulated by microtubule-associated proteins (MAPs) and plus-end tracking proteins (+TIPs) that accumulate at growing MT plus ends [1, 2]. End-Binding protein EB1 is a +TIP that plays a pivotal role in orchestrating protein interaction networks at growing MT ends. EB1 binds MTs with its N-terminal calponin homology (CH) domain and displays in its C-terminal portion an EB homology (EBH) domain responsible for interaction with a wide variety of regulatory +TIPs. EB1-recruited proteins contain either a cytoskeleton-associated protein glycine-rich domain (CAP-Gly) or a consensus sequence SxIP (serine - any aminoacid - isoleucine - proline) embedded in an intrinsically unstructured polypeptide region rich in basic, proline and serine residues [2-6]. In addition to its role as a molecular platform for regulatory +TIPs, EB1 has an intrinsic regulatory effect on MT dynamics at growing ends. EB1 senses the nucleotide state of MTs and is able to bind autonomously an extended GTP/GDP-Pi cap structure at the MT end [7-9] with more than 10-fold higher affinity compared to the microtubule lattice [8]. Measurements of EB1 protein dynamics showed that they exchange very rapidly at growing MT ends with fast binding/unbinding kinetics [10-12]. In mammalian cells [13] and Xenopus extracts [10], EB1 has been shown to increase persistent MT growth and suppress catastrophe frequency. Recent *in vitro* studies have identified EB1 as a MT maturation factor that decreases the maturation time of growing MT ends [14], providing a mechanistic link between EB1 localization and regulation of MT dynamics. However, negative regulation of EB1 association with MT growing ends, which is essential to EB1 function, remains poorly understood.

ATIP3 is a novel MAP encoded by candidate tumor suppressor gene *MTUS1* whose expression is markedly down-regulated in a variety of human cancers [15-17]. ATIP3 re-expression at normal levels into breast cancer cells significantly reduces cell proliferation, tumor growth and metastatic dissemination in animal models [15, 17] underlying important tumor suppressor effects. ATIP3 also limits cell migration by decreasing cell polarity and directionality, and impairs the ability of MTs to reach the cell cortex as a consequence of reduced MT dynamics at the plus ends [17]. Conversely, ATIP3 depletion increases MT dynamic instability by increasing MT growth and growth rate, and decreasing catastrophe frequency and time spent in attenuated state [17]. Interestingly, the effects of ATIP3 deficiency on MT dynamic instability parameters are superimposable to those observed upon EB1 expression in living cells, leading us to investigate whether ATIP3 may negatively regulate EB1 functions at growing MT ends.

In the present study, we show that ATIP3 interacts with EB1 in an MT-independent manner. The interaction involves a non-canonical sequence that directly binds EB1 *in vitro*. ATIP3-EB1 complexes are present in the cytosol and impair EB1 accumulation at growing MT ends. FRAP analyses indicate that ATIP3 deficiency increases the dynamic exchange of EB1 at growing ends with no modification of EB1 diffusion in the cytosol. Our results support a novel model for negative regulation of EB1 turnover at MT plus ends.

## RESULTS AND DISCUSSION

### ATIP3 interacts with EB1

**Figure 1:**
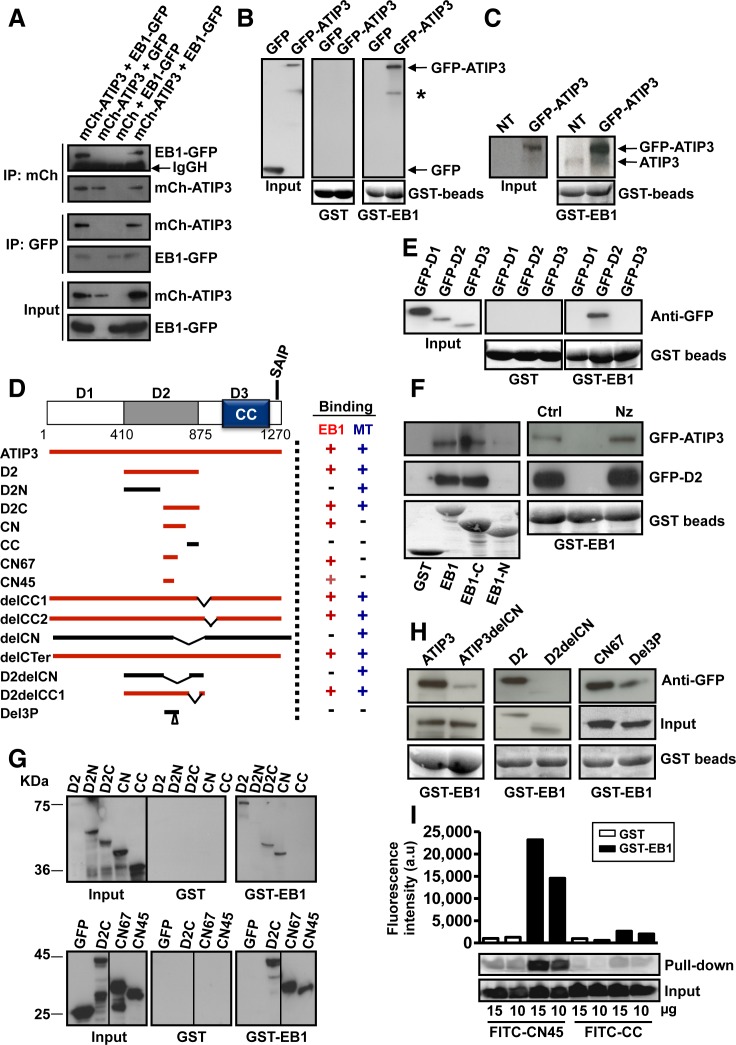
ATIP3 interacts with EB1 **A.** MCF7 cells were transfected with mCh-ATIP3 (or mCh) and EB1-GFP (or GFP) and immunoprecipitation was performed using anti-GFP or anti-mCh antibodies as indicated. Western blots were probed with anti-MTUS1 and anti-GFP antibodies to reveal mCh-ATIP3 (210 KDa) and EB1-GFP (55 KDa). IgGH: Immunoglobulin heavy chain. **B.** GST pull-down assays of MCF7 cell lysates transiently expressing GFP-ATIP3 or GFP using GST or GST-EB1 beads as indicated. Blots were probed with anti-GFP antibodies. An asterisk indicates cleavage product of GFP-ATIP3. Lower panel: GST beads loading. **C.** Purified GST-EB1 beads were used to precipitate GFP-ATIP3 (210 KDa) and endogenous ATIP3 (180 KDa) expressed in HeLa cells. Blots were probed with anti-MTUS1 antibodies. Lower panel: GST beads loading. **D.** Schematic representation of ATIP3 protein illustrating the position of D1, D2 and D3 regions. CC: coiled coil region. Position of the SAIP motif (residues 1249-1252) is shown. Amino acid numbering is from Accession number NP_001001924. Lower panel: Schematic drawing of ATIP3 deletion mutants and their ability (+) or not (−) to bind EB1 and MTs. **E.** GST-pull-down assays of MCF7 cell lysates expressing GFP-fused D1, D2, D3 regions as in B. **F.** Pull-down assays of MCF7 cell lysates expressing GFP-ATIP3 or GFP-D2, using GST-EB1, GST-EB1-C and GST-EB1-N as indicated (left panel) or following treatment with 10 μM nocodazole (Nz) or vehicle (Ctrl) for 1h at 4°C (right panel). Blots were probed as in B. **G.** Pull-down analysis of GFP-D2 fragments using GST and GST-EB1 beads as in B. Molecular weights (KDa) are on the left. **H.** Pull-down analysis of ATIP3 deletion mutants using GST-EB1. Blots were probed with anti-GFP antibodies as in B. Lower panel : GST beads loading. **I.** *In vitro* pull-down assay using GST (white bars) or GST-EB1 (black bars) beads and 10 or 15 μg of synthetic fluorescent CN45 and CC peptides as indicated below. Upper panel: fluorescence intensity (arbitrary units) in the precipitates. Lower panel: fluorescence was detected in samples before (input) and after GST pull-down, using Typhoon scanner following resolution on 15%SDS-PAGE.

To investigate whether ATIP3 interacts with EB1, we used anti-mCherry (mCh) antibodies to isolate mCh-ATIP3 complexes from MCF7 cells expressing mCh-ATIP3 fusion protein and EB1 fused to green fluorescent protein (EB1-GFP). Western blotting with anti-GFP antibodies confirmed the presence of a mCh-ATIP3-EB1-GFP complex (Figure 1A, upper panel). In a reciprocal experiment, mCh-ATIP3 was detected by anti-mCh antibodies following immunoprecipitation of EB1-GFP (Figure 1A, middle panel) as well as GFP-EB3 and EB3-GFP (Supplementary Figure S1A), indicating that ATIP3 is able to bind EB1 and EB3 in intact cells. Of note, ATIP3-EB1 complexes were not detectable by co-immunoprecipitation of endogenous proteins expressed in HeLa cells, suggesting that the interactions between these proteins are weak or transient and/or involve a fraction of the proteins.

GST pull-down assays were performed using GST-EB1 as an affinity matrix. As shown in Figure 1B, GST-EB1, but not GST, precipitated GFP-ATIP3 fusion proteins as well as endogenous ATIP3 expressed in HeLa cells (Figure 1C). Full-length ATIP3 was then cleaved into three regions designated D1, D2 and D3 (Figure 1D) that were fused to GFP. Pull-down experiments using GST-EB1 (Figure 1E) revealed that only the central (D2) region is retained on GST-EB1 beads. Of note, the C-terminal D3 region does not interact with EB1 although it contains a bona fide SxIP motif embedded in an intrinsically unstructured polypeptide region rich in basic, proline and serine residues (Supplementary Figure S1B), which is a hallmark of EB1 binding [3, 5, 18]. Accordingly, deleting the last 30 residues of ATIP3 (DelCTer mutant) did not abrogate EB1 binding (Supplementary Figure S1C). Furthermore, EB1 binding was not affected by replacement of core hydrophobic residues of the consensus SAIP motif with polar residues (Ile and Pro at positions 1251-1252 of the ATIP3 sequence changed to Asn and Ala, respectively) in SANP and SAIA mutants (Supplementary Figure S1C), further indicating that the canonical SxIP motif of ATIP3 is not essential to EB1 interaction.

To map the domain of EB1 that interacts with ATIP3, we used GST-EB1 deletion mutants comprising either the N-terminal (EB1-N) or C-terminal (EB1-C) portion of the molecule [20]. As shown in Figure 1F (left panel), both ATIP3 and D2 interact with the C-terminal region of EB1, as do other EB1 partners [1-6]. Of note, D2 still binds GST-EB1-ΔAc (Supplementary Figure S1D), an EB1 deletion mutant that lacks the last 19 amino acids and does not interact with SxIP-containing proteins [5, 19], suggesting that the mode of interaction between EB1 and ATIP3 differs from that of EB1-SxIP partners.

To evaluate whether MTs may contribute to ATIP3-EB1 binding, cells were treated with nocodazole at a concentration that totally depolymerizes cellular MTs. In these conditions, ATIP3 and D2 still bound EB1 (Figure 1F, right panel), indicating that EB1-ATIP3 interaction does not require an intact MT network.

To map further the EB1-interacting region of ATIP3, a series of deletion mutants (Figure 1D) were analyzed in GST-EB1 pull-down experiments. As shown in Figure 1G (upper panel) D2C and CN fragments retained the ability to interact with EB1. Deletion of the CN sequence in ATIP3 and D2 polypeptides (ATIP3delCN and D2delCN mutants) led to a marked decrease in EB1 interaction (Figure 1H) whereas deleting the adjacent CC sequence (delCC1, delCC2, D2delCC1 mutants) did not abrogate EB1 binding (Supplementary Figure S1E). The EB1-interacting region was further refined to sequences of 67 and 45 amino acids (CN67 and CN45, respectively) (Figure 1G, lower panel). The minimal EB1-interacting sequence CN45 is evolutionary conserved (Supplementary Figure S1F) and includes a proline-rich motif whose deletion (Del3P mutant) markedly reduced EB1 binding (Figure 1H).

To investigate whether the interaction is direct, a fluorescent peptide corresponding to the CN45 sequence was synthesized and analyzed in GST-EB1 pull-down assays *in vitro*. The CC sequence, that does not bind EB1, was used as a negative control. As shown in Figure 1I, FITC-CN45 (but not FITC-CC) was specifically retained on GST-EB1 beads, indicating direct interaction between CN45 and EB1 and suggesting that ATIP3 might bind EB1 directly. Altogether, these results demonstrate that ATIP3 interacts with EB1 *via* a non-canonical motif present in the C-terminal part of the D2 sequence.

### EB1-interaction and MT-binding involve different ATIP3 regions

**Figure 2:**
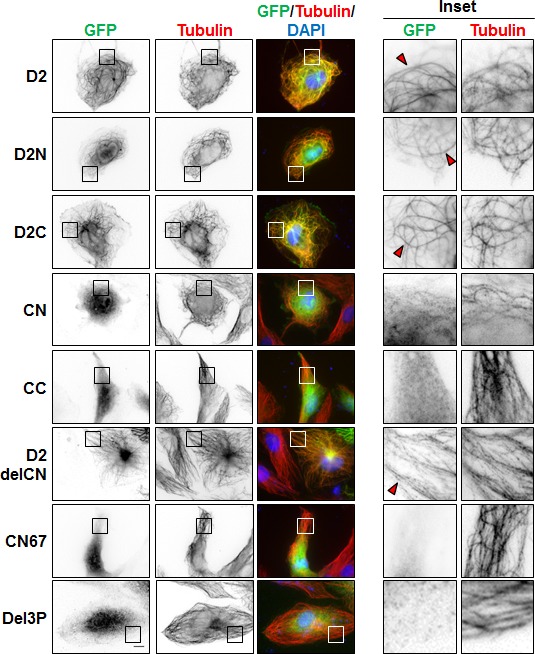
Cellular localization of GFP-D2 and deletion mutants Immunofluorescence imaging of RPE-1 cells expressing GFP-D2 and GFP-tagged deletion mutants as indicated. Cells were fixed and stained with anti-GFP (green), anti-alpha-tubulin (red) antibodies and DAPI (blue). Enlarged portions of the selected areas are shown in the insets. Red arrowheads show MT-localization of GFP-fusion proteins. Scale bar, 10μm.

To evaluate whether EB1-interaction and MT-binding may involve the same region of ATIP3, we analyzed the cellular localization of GFP-fused D2 deletion mutants by immunofluorescence. As shown in Figure 2, both N-terminal (D2N) and C-terminal (D2C) portions of D2 co-localized with tubulin along the MT lattice. Shorter deletion mutants of D2C (CN and CC) remained mostly cytosolic, suggesting that MT localization involves a conformational recognition motif that requires both parts of the sequence. Importantly, the EB1-interacting domain CN was diffuse in the cytosol whereas the D2delCN deletion mutant, that has lost EB1 binding, still decorated the MT lattice (Figure 2, Supplementary Table S1), indicating that EB1-interaction is independent of MT-binding and that the two interacting regions are not overlapping.

### ATIP3-EB1 complexes are present in the cytosol

**Figure 3:**
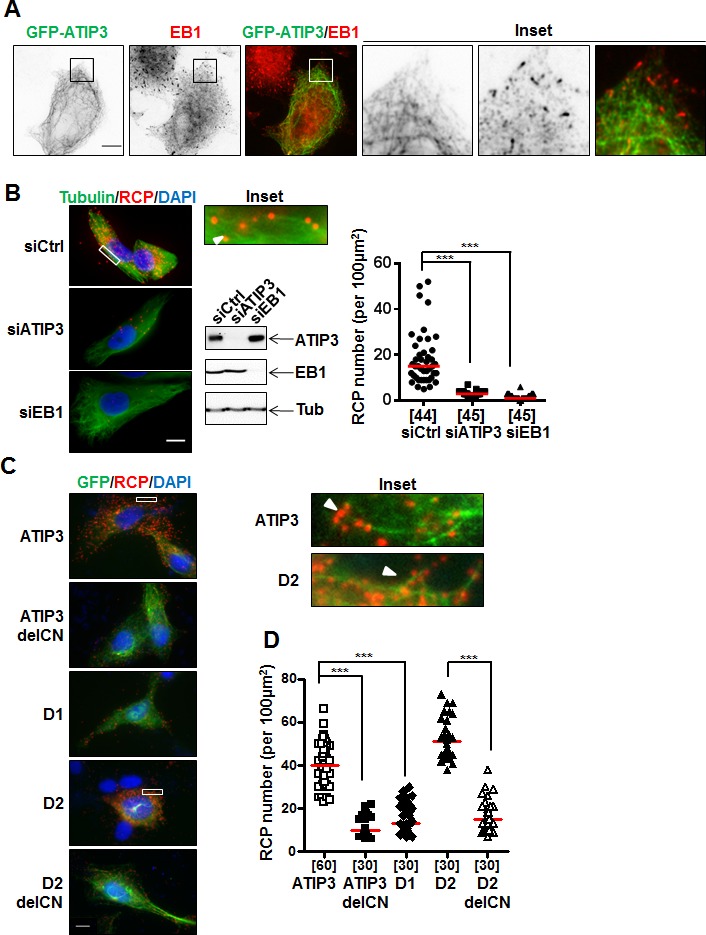
*In situ* interaction between ATIP3 and EB1 **A.** RPE-1 cells transfected with low amounts of GFP-ATIP3 were fixed and stained with anti-GFP (green) and anti-EB1 (red) antibodies. Insets show that EB1 comet-like structures at plus ends are not stained by GFP-ATIP3. Magnification 100X. Scale bar 10μm. **B.** PLA performed in HeLa cells transfected with control (siCtrl), ATIP3-specific (siATIP3) or EB1-specific (siEB1) siRNA as indicated. Silencing efficiency was assessed by immunoblotting with anti-MTUS1 (ATIP3), anti-EB1 and anti-tubulin (Tub) antibodies. Molecular proximity between endogenous ATIP3 and EB1 was analyzed using rabbit anti-MTUS1 and mouse anti-EB1 primary antibodies and revealed as red signals of RCP stained with cy5-labeled oligonucleotide probe. Cells were stained with anti-alpha-tubulin antibodies to reveal the MT network. Shown are merge pictures of tubulin (green), RCP (red) and nuclei (blue) staining. Scale bar 10μm. Inset: enlarged portion of a selected area from siCtrl-transfected cell. The arrowhead illustrates alignment of RCP signals along the MT lattice. Right panel: quantification of the number of RCP per area (100 μm^2^). Number of areas analyzed (5 areas from at least 6 different cells in 3 independent experiments) is under brackets. ***p < 0.0001. **C.** PLA performed in RPE-1 cells transfected with GFP-tagged ATIP3 regions and deletion mutants as indicated on the left, using rabbit anti-GFP and mouse anti-EB1 primary antibodies. Shown are merge pictures of GFP (green), RCP (red) and nuclei (blue) staining. Scale bar 10μm. Inset: enlarged portions of selected areas. Arrowheads illustrate alignment of RCP signals along the MT lattice. **D.** Quantification of the number of RCP per area (100 μm^2^) as in **B.** Five areas from at least 6 single cells in 3 independent experiments were analyzed. ***p < 0.0001.

The interaction between ATIP3 and EB1 prompted us to examine whether EB1 may recruit ATIP3 at growing MT plus ends. We used RPE-1 cells that have a sparse MT array and are well suited for visualizing individual MTs and MT ends [17]. Cells were transfected with levels of GFP-ATIP3 close to endogenous, to avoid MT bundling due to ATIP3 overexpression [17]. As shown in Figure 3A, EB1 comet-like structures were still detectable in low GFP-ATIP3-expressing cells and GFP-ATIP3 was distributed along the MT lattice but did not co-localize at MT ends together with endogenous EB1. Time-lapse analysis (Supplementary Figure S2A, Movies 1 and 2) also clearly showed distinct patterns of mCh-ATIP3 and EB3-GFP localization in living cells and indicated that ATIP3 does not accumulate at growing MT ends. Finally, time-lapse images of MCF7 cells stably expressing moderate levels of GFP-ATIP3 (Supplementary Figure S2B, Supplementary Movie 3) confirmed that ATIP3 decorates the MT lattice and has no tip-tracking properties. Interestingly, they also revealed for the first time that ATIP3 accumulates at the end of shrinking microtubules in living cells, highlighting its back-tracking behavior.

To reveal the location of ATIP3-EB1 complexes inside the cells, we used the Proximity Ligation Assay (PLA) duolink technology that allows *in situ* detection of molecular complexes in single cells at the location where the proteins of interest interact [21]. Molecular proximity between endogenous ATIP3 and EB1 proteins was assessed in HeLa cells using anti-MTUS1 and anti-EB1 primary antibodies followed by *in situ* detection of fluorescent Rolling Circle amplification Products (RCP). Individual bright fluorescent RCP signals were detected in control cells but not following transfection with ATIP3- or EB1-siRNA (Figure 3B). Red RCP signals indicating molecular proximity between endogenous ATIP3 and EB1 proteins were distributed throughout the cytosol (Figure 3B) and were found to some extent to colocalize with the MT lattice (Figure 3B, inset).

PLA amplification signals were also detected using anti-GFP and anti-EB1 primary antibodies in RPE-1 cells expressing GFP-ATIP3 and GFP-D2, but not GFP-D1 (Figure 3C) nor in negative control conditions (Supplementary Figure S3A, S3B and S3C). Specific PLA signals were also revealed in mCh-ATIP3-transfected RPE-1 cells stably expressing EB1-GFP using anti-mCh and anti-GFP primary antibodies (Supplementary Figure S3D). In line with GST-EB1 pull-down assays, PLA analyses revealed the presence of *in situ* molecular complexes between endogenous EB1 and GFP-D2C and GFP-CN, but not GFP-D2N and GFP-CC regions (Supplementary Figure S3E). The number of *in situ* PLA amplification signals was markedly reduced in cells transfected with ATIP3delCN and D2delCN deletion mutants as compared to full-length ATIP3 and D2 region (Figure 3D), further demonstrating the involvement of the CN sequence in EB1-ATIP3 complex formation in intact cells.

### ATIP3 interacts with EB1 to limit its accumulation at growing ends

**Figure 4:**
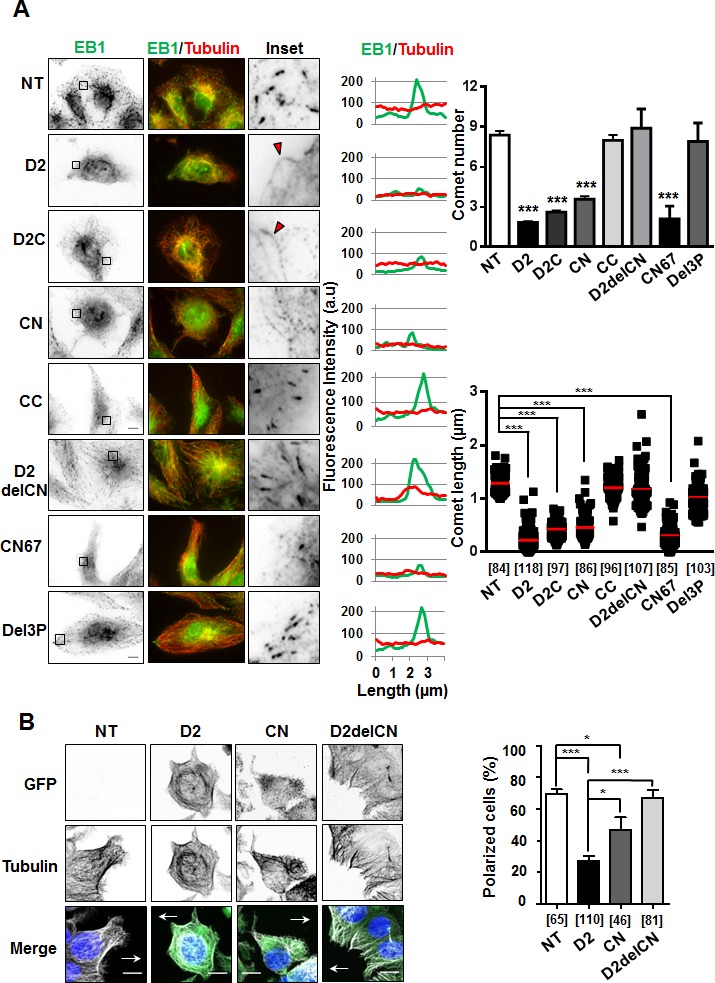
Effect of deletion mutants on EB1 comet formation and cell polarity **A.** Anti-EB1 (green) and anti-tubulin (red) immunostaining of RPE-1 cells transiently transfected with GFP-D2 and GFP-tagged regions. Insets: EB1 comet-like structures in enlarged portions of selected areas. Red arrowheads illustrate weak EB1 staining along the MT lattice. Right panels show distribution of EB1 (green) and alpha-tubulin (red) at the microtubule plus ends (linescans), and quantification of number of comets (per 62μm^2^ area) and comet length (scattered dot plot) from 5 single cells in 3 to 5 independent experiments. Number of comets analyzed is under brackets. NT: non-transfected. Scale bar, 10μm. ***p < 0.0001. **B.** Immunofluorescence imaging of migrating MCF-7 cells transfected with GFP constructs as indicated. Cells were allowed to polarize for 3 hrs, then fixed and stained with anti-GFP (green), anti-α-tubulin (white) antibodies and DAPI (blue). Arrows indicate the direction of migration. Right panel shows quantification (percent) of polarized MCF-7 cells from 3 to 6 fields, in 2 independent experiments. Number of cells analyzed is under brackets. Scale bar, 10μm. *p < 0.05; ***p < 0.0001.

To investigate the consequence of ATIP3-EB1 interaction on the accumulation of EB1 as comet-like structures at growing MT ends, which is a hallmark of MT dynamics, we analyzed EB1 staining in the presence of D2 and deletion mutants. As shown in Figure 4A, expression of EB1-interacting regions D2C and CN reduced the number and length of EB1 comets to the same extent as D2, whereas CC - that does not bind EB1 - had no significant effect on EB1 comet-like structures. Of importance, deletion mutants (D2delCN and Del3P) having lost the ability to interact with EB1 were no longer able to reduce EB1 accumulation at growing MT ends (Figure 4A, Supplementary Table S1), highlighting functional relevance of ATIP3-EB1-interaction.

To validate our results, rescue experiments were performed on ATIP3-silenced HeLa cells in which GFP-ATIP3 was re-introduced at levels close to endogenous (Supplementary Figure S4A). As expected, EB1 comets in ATIP3-depleted cells were significantly increased as compared to control, and moderate levels of GFP-ATIP3 were sufficient to fully rescue the ATIP3 knock-down phenotype (Supplementary Figure S4B). Importantly, moderate expression of GFP-D2 and GFP-CN, but not GFP-D2delCN, in ATIP3-silenced HeLa cells was sufficient to restore EB1 comets (Supplementary Figure S4C, S4D and S4E) further confirming that ATIP3/EB1-interaction is essential to the regulatory effects of ATIP3 on EB1 comets formation.

We then investigated the consequence of ATIP3 silencing on the localization of EB1 partners such as CLIP-170 and the MCAK kinesin, that bind EB1 *via* a CAP-Gly and a SxIP motif, respectively [1, 2]. As shown in Supplementary Figure S5A and S5B, the number of GFP-CLIP-170 and GFP-MCAK comets that co-localized with EB1 was increased in ATIP3-depleted cells, indicating that ATIP3 reduces the accumulation of EB1/+TIPs molecular complexes at growing MT ends. Of note, a MCAK-3E mutant that does not interact with EB1 [3] remained cytosolic in both control and ATIP3-silenced cells (Supplementary Figure S5C), confirming that regulation of MCAK comets by ATIP3 requires EB1. Thus, by interacting with EB1, ATIP3 negatively regulates the association of EB1 molecular complexes at MT growing ends. This, in turn, may contribute to fine-regulation of MT dynamics in the presence of ATIP3.

As a consequence of decreased MT dynamics, the ability of MT tips to reach the cell cortex was markedly impaired during cell migration, leading to defects in cell polarity in migrating D2-positive cells compared to control cells (Figure 4B). Deletion of the EB1-interacting domain (D2delCN mutant) restored a radial array of MTs at the cell margin and abrogated the effect of D2 on cell polarity, further illustrating the importance of EB1 interaction in ATIP3 function. Of interest, expression of the EB1-binding region CN partially impaired MT organization and cell polarity (Figure 4B) but was not sufficient to fully recapitulate the effects of ATIP3 suggesting that other ATIP3 regions, possibly involved in MT binding, might also contribute to ATIP3 biological function.

### ATIP3 silencing increases EB1-GFP exchange on MT ends

**Figure 5:**
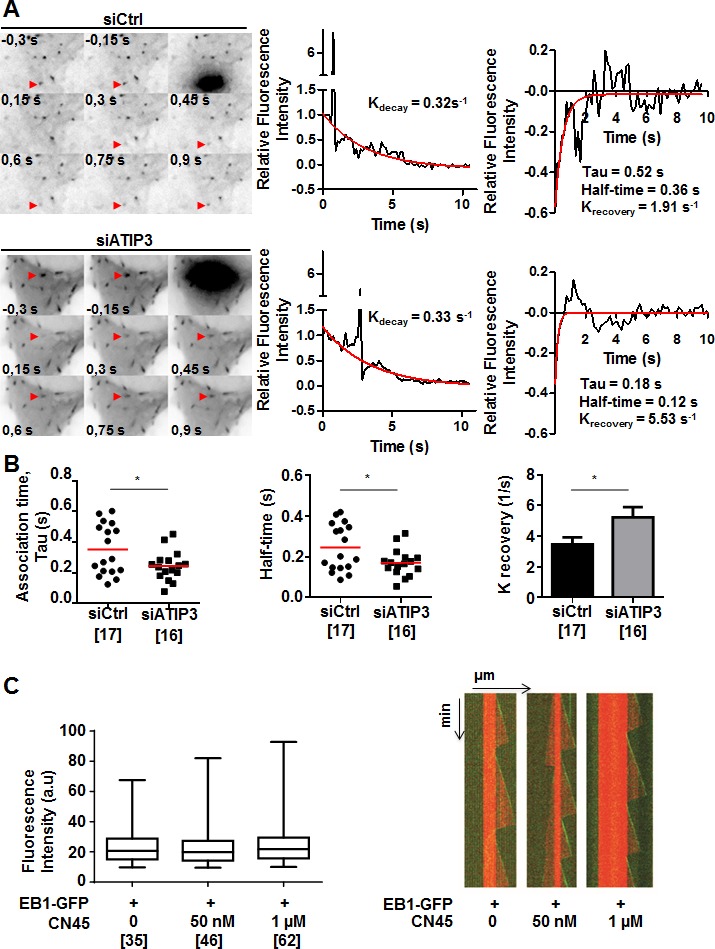
ATIP3 silencing increases EB1-GFP exchange on MT ends **A.** Representative example of time lapse imaging of HeLa cells expressing EB1-GFP. Fluorescence was bleached at the distal part of the MT in control (siCtrl, upper) and ATIP3-silenced (siATIP3, lower) cells. Red arrowhead indicates position of the bleached comet. Fluorescence intensity of the bleached comet (middle panel) and fluorescence recovery curves (right panel) are shown. Red line in middle and right panels indicates fluorescence decay and recovery, respectively. **B.** Quantification of association time Tau in seconds (s), half-time of recovery in seconds (s), and k_recovery_ (s-^1^) of fluorescence recovery in control cells (*n* = 17) and ATIP3-depleted cells (*n* = 16) in 2 independent experiments. Number of EB1-GFP bleached comets analyzed is under brackets. **C.** Box-n-Whisker plot showing fluorescence intensity of EB1-GFP at microtubule plus ends in the presence of purified EB1-GFP (100 nM) and increasing amounts of CN45 peptide as indicated. Figure was generated with PRISM 6.0. Shown is one representative experiment out of 3. Number of comets appearing over 30 minutes is under brackets. Right panel : Kymographs representing MT assembly in the presence of tubulin (15 μM), EB1-GFP (100 nM) and increasing amounts of CN45 peptide. Tubulin and EB1-GFP are detected by fluorescence (tubulin in red and EB1-GFP in green). Shown is one representative out of 3 independent experiments.

To get more insight into the mechanism by which ATIP3 negatively regulates EB1, we examined the consequence of ATIP3 depletion on the dynamic association of EB1 with MT plus ends. Fluorescence recovery after photobleaching (FRAP) experiments were performed on HeLa cells expressing EB1-GFP following transfection with either control or ATIP3-specific siRNA. Bleaching was done in the distal part of growing MT ends and recovery of EB1-GFP fluorescence was measured (Figure 5A, Supplementary Movie 4). After correction for cytoplasmic background and fluorescence decay, K recovery for the exchange of EB1-GFP on MT ends in control cells was 3.47±0.45 s^−1^ (half-life of association 0.24±0.03 s), which is very close to values previously reported for EB3-GFP recovery on MT ends in COS-7 cells (K recovery of 3.37 s^−1^, half-life of association 0.20 s) [12]. As shown in Figure 5A and 5B, EB1-GFP fluorescence recovery at MT plus ends was significantly faster in ATIP3-silenced cells (K recovery of 5.23±0.66 s^−1^, half-time of association 0.17±0.02 s), with no significant modification of EB1-GFP fluorescence decay (Supplementary Figure S6A), indicating that EB1 exchange on its recognition site at growing MTs is increased in the absence of ATIP3. Of note, the lifetime of EB1-GFP labeled region on MT plus ends (EB1 decoration time, that specifies EB1-interacting sites at MT ends) remained unchanged in ATIP3-deficient *versus* control cells (2.68 +/− 0.93 (*n* = 100) and 2.67 +/− 1.28 (*n* = 70), respectively) (Supplementary Figure S6B), indicating that ATIP3 silencing does not alter EB1-recognition sites on MT ends.

To investigate whether ATIP3 may impair EB1 free diffusion in the cytosol, which is rate limiting for EB1 exchange at MT plus end [11, 12], EB1-GFP fluorescence recovery after photobleaching was measured in the cytosol of HeLa cells following ATIP3-silencing. As shown in Supplementary Figure S6C and S6D, ATIP3 depletion does not significantly modify EB1-GFP fluorescence recovery in the cytosol (K recovery of 2.2 ± 0.3 s-1 in control cells *versus* K recovery of 2.1 ± 0.6 s-1 in ATIP3 silenced HeLa cells), ruling out a major effect of ATIP3 on EB1 mobility. Altogether, these results indicate that ATIP3 binds EB1 in the cytosol to indirectly slower its binding/unbinding kinetics on MT plus ends, without affecting free diffusion of EB1.

*In vitro* studies were conducted to further explore the consequence of ATIP3-EB1 interaction in a cell-free system. Using dual-color time-lapse total internal reflection fluorescence (TIRF) microscopy, we analyzed fluorescence intensity of purified EB1-GFP in the presence of dynamic ATTO-565 labeled microtubules and increasing amounts of CN45 peptide, that directly binds EB1 *in vitro*. As shown in Figure 5C, CN45 even in high molar excess had no significant effect on EB1 comets intensity, indicating that ATIP3-EB1 interaction *in vitro* is not sufficient to regulate EB1 localization at growing MT ends. Accordingly, MT dynamic instability parameters measured *in vitro* were not modified in the presence of EB1-interacting peptide CN45 (Figure 5C, Supplementary Table S2). Together, these studies suggest that negative regulation of EB1 at the MT plus ends by ATIP3 may require the presence of additional cellular components or involve post-translational modifications.

### A novel model for negative regulation of EB1 at growing MT ends

Our data support a model in which cytosolic ATIP3-EB1 complexes slower the dynamic association of EB1 on growing MT ends, without affecting EB1 free diffusion and with no major alteration of its recognition site. In ATIP3-silenced cells, EB1 turnover is faster and EB1 is more prone to accumulate at plus ends together with its interacting partners, therefore leading to increased MT dynamic instability and subsequent increase in cell polarity.

Recent studies conducted in developing neurons have shown that MAP1B and tau, two stabilizing MAPs that decorate MTs, also interact with EB1/3 and negatively regulate their accumulation at growing MT ends [22, 23]. A sequestration model has been proposed in which EB1 is retained in the cytosol by interaction with MAP1B [22] or immobilized by elevated levels of tau proteins [23]. Of note, quantitative proteomics studies performed in NIH-3T3 [24], UO2S [25] and HeLa [26] human cell lines have revealed high EB1 protein copy number (4.10^5^ to 1.10^6^ molecules per cell) compared to ATIP3 (from undetectable to 300 or 1000 times less abundant than EB1), making it unlikely that ATIP3 sequesters EB1 in the cytosol. Arguing against a sequestration mechanism, FRAP analyses of EB1-GFP recovery indicate that ATIP3 silencing does not modify free mobility of EB1 in the cytosol. Our *in vitro* studies, showing that the purified CN45 peptide has no significant effect on EB1 comets intensity, further rule out the possibility that direct ATIP3-EB1 interaction may be responsible for decreased EB1 comets at MT ends. Together, our findings favor a novel mechanism by which ATIP3 interacts with EB1 in the cytosol and indirectly slowers its turnover on growing MT ends, thereby leading to decreased MT dynamics and cell polarity.

Given the potent tumor suppressor effects of ATIP3, these findings may have clinical relevance in the field of cancer. Indeed, EB1 overexpression has been reported in hepatocellular carcinoma [27], breast cancer [28], colorectal carcinoma [29, 30] and glioblastoma [31] and is associated with tumor progression and reduced survival of the patients [27, 31]. Decreasing EB1 association with its binding site at MT ends, with subsequent decrease in MT dynamics and cell polarity, may thus represent an interesting therapeutic option. Among the wide variety of EB1 partners, only few have been recognized as tumor suppressors [5]. The major tumor suppressor Adenomatous Polyposis Coli (APC) is a well-known EB1 interacting protein that paradoxically cooperates - rather than competes - with EB1 during mitosis and cell migration [32-34]. The Cylindromatosis CYLD tumor suppressor is another EB1-interacting +TIP that also acts in concert with EB1 to regulate MT dynamics and cell migration [35]. Results presented here provide the first evidence for negative regulation of EB1 dynamic association at MT ends by cytosolic interaction with a tumor suppressor protein. This study extends our knowledge of EB1 regulation at growing MT ends and may have important implications in the field of cancer, in particular for aggressive tumors showing ATIP3 deficiency.

## MATERIALS AND METHODS

### Cell lines

Human breast cancer cell lines MCF7, HC7 (MCF7 cells stably expressing endogenous levels of GFP-ATIP3) and D3H2LN, as well as SV-MRC5 lung fibroblasts, HeLa and RPE-1 cells were described previously [15, 17]. MCF7, SV-MRC5-SV and RPE-1 cells express undetectable levels of ATIP3, whereas HeLa cells express endogenous ATIP3 [15, 17]. Cells were routinely authenticated by morphologic observation and tested for absence of mycoplasma contamination using MycoAlert Assay detection kit (Lonza, France).

### Plasmids constructs and transfections

Plasmids encoding GFP-ATIP3, mCh-ATIP3, GFP-D1, GFP-D2 and GFP-D3 were described elsewhere [17]. GFP-fused D2 fragments and deletion mutants were obtained by PCR-amplification of ATIP3 sequence or Site-Directed Mutagenesis using oligonucleotides shown in Supplementary Table S3. Expression vectors encoding GST-EB1, GST-EB1-C, GST-EB1-N and GST-EB1-ΔAc were gifts of Dr Anna Akhmanova (Utrecht University, The Netherlands). EB1-GFP and GFP-CLIP-170 constructs were provided by Dr Franck Perez (Institut Curie, Paris, France). GFP-MCAK and GFP-MCAK-3E were kindly provided by Dr Michel Steinmetz (Paul Scherrer Institute, Switzerland). All cDNA constructs were transfected (1 to 2 μg) for 24h using Dreamfect (Oz Biosciences) or X-treme Gene 9 (Roche).

ATIP3-specific siRNA [15], EB1-specific siRNA (on-target plus smart pool, NM_012325) and scrambled siRNA were from Dharmacon (ThermoFisher Scientific). All siRNAs (50 nM) were transfected using lipofectamine 2000 (Invitrogen) and silencing efficiency was evaluated by immunoblotting using rabbit anti-MTUS1 antibodies (ARP44419; Aviva Systems, CA, USA) and rat anti-EB1 (KT51; Santa Cruz).

### GST pull-down assays and immunoprecipitations

Purification of GST fusion proteins and GST pull-down assays from extracts of HeLa cells or MCF7 transiently expressing GFP fusion proteins were performed as described [3, 20] using 15-30 μg of GST or GST-EB1 beads per experimental condition. Immunoblotting was using rabbit anti-MTUS1 (ARP44419, Aviva Systems) (1:1000) or monoclonal anti-GFP antibodies (clone 7.1/13.1, Roche) (1:3000).

For *in vitro* interaction, chemically synthesized peptides CN45 and CC coupled to FITC were purchased from GL Biochem (Shanghai, China). Purified peptides were incubated for 1h at room temperature with GST- or GST-EB1 beads in 50mM HEPES containing 150mM NaCl, 0.01% Triton X100 (pH 7.4) then washed in the same buffer. Interaction was assessed by FITC fluorescence measurement using Fusion Universal Microplate Analyzer (Packard BioScience) and with Typhoon™ system (Amersham Biosciences) following 15% SDS-PAGE.

For immunoprecipitation, cell lysates were incubated for 2h at 4°C with 4μg of mouse monoclonal anti-GFP (Roche), or mouse monoclonal anti-mCh (Clontech) antibodies prior to incubation with G protein-sepharose beads. Bound proteins were detected by immunoblotting as described above.

### Immunofluorescence and live cell imaging

Transfected RPE-1 cells were fixed with ice-cold methanol and incubated as described with human anti-alpha-tubulin clone F2C [17], rat anti-EB1 (Santa Cruz) or mouse anti-GFP (Roche). Linescan analyses of fluorescence intensity were performed on a 5 μm line along the length of MT plus end [17]. Results shown are representative of 3 to 5 independent experiments. For quantification of comet number, 5 different areas of at least 5 single cells were analyzed in 3 to 5 independent experiments.

For live cell imaging, SV-MRC5 co-transfected for 24h with mCh-ATIP3 and EB3-GFP were imaged by spinning disc confocal microscopy as described in Supplementary Methods. Images were acquired in a stream mode at 500 ms exposure time.

For backtracking experiments, HC7 [17] were imaged using TIRF videomicroscopy as described in Supplementary Methods. Images were acquired in a stream mode at 100 ms exposure time.

### TIRF microscopy for *in vitro* studies

Perfusion chambers were functionalized with silane-PEG-biotin (Laysanbio) coverslips and silane-PEG (Creative PEGwork) glass slides as described [36]. The flow cell was firstly perfused with neutravidin (25 μg/ml in 1% BSA/BRB80 (80 mM PIPES, 1 mM GTP, 1 mM MgCl_2_, 1 mM EGTA, pH 6.75) and secondly with GMPCPP-stabilized, ATTO-565 labelled microtubule seeds. Microtubules assembly was performed at 32°C from 15 μM brain tubulin (containing 20% ATTO 565-labeled tubulin) in the presence of 100 nM EB1-GFP and 0, 0.05 μM or 1 μM CN45 peptide (GL Biochem, Shanghai, China) in BRB80 buffer supplemented with 4 mM DTT, 1% BSA, 50 mM KCl, 1 mg/ml glucose, 70 μg/ml catalase, 580 μg/ml glucose oxydase, 0.1 % methylcellulose (4,000 centipoise). Dual-color time-lapse imaging was performed on an inverted Eclipse Ti (Nikon) microscope with an Apochromat 60×1.49 N.A oil immersion objective (Nikon), equipped with an ilas2 TIRF system (Roper Scientific), a cooled charged-coupled device camera (EMCCD Evolve 512, Photometrics) and controlled by the MetaMorph 7.7.5 software (Molecular Devices). For excitation we used 491- and 561-nm lasers. Time-lapse imaging was performed at 1 frame per 2 s with an 80-ms exposure time, during 30 minutes.

The image analysis was performed with ImageJ software, version 1.43u (W. Rasband, NIH. USA). The comet fluorescence intensities were measured over the tubulin polymerization periods using kymographs with background subtraction (protocol based on [8]). The average fluorescence intensity at microtubule growing ends was measured in the green channel with a 5-pixel-wide line. Only comet fluorescence intensity differences superior to one background standard deviation were included in the calculation. Comparisons of fluorescence intensities with and without CN45 were calculated from experiments performed with the same acquisition set-up and laser intensity. Microtubule dynamic parameters were determined on kymographs using Image J software, version 1.43u (W. Rasband, NIH. USA). Statistical analyses were done with Prism 6.0 (GraphPad software, USA).

### Fluorescence recovery after photobleaching (FRAP) analysis

HeLa cells were co-transfected with appropriate siRNA (72h) and EB1-GFP (24h). FRAP analyses were performed after bleaching MT ends, or selected cytosolic regions, and recovery was measured as described in Supplementary informations. To calculate the recovery of EB1-GFP fluorescence after photobleaching, the fit of the fluorescence decay was subtracted to the normalized curve to obtain a curve with only the mean fluorescence recovery. This curve could be fitted with the first-order exponential decay with a robust fitting routine: y = (y_0_ − plateau) * exp^(−krecovery*x)^ + plateau, where y is normalized intensity, y_0_ is the initial fluorescence value, plateau is the y value at infinite times, (y_0_ − plateau) is the span of the reaction, k_recovery_ is the reaction constant and x is the time. Half-time of recovery was calculated as ln(2)/k_recovery_. Association time (Tau) was calculated as 1/k_recovery_.

### Proximity ligation assay (PLA)

*In situ* PLA detection was carried out using DUOLINK II *In Situ* Far Red kit (Sigma-Aldrich, St Louis, USA). HeLa cells were transfected (72h) with appropriate siRNAs, fixed and incubated with rabbit anti-MTUS1 (ARP44419, Aviva Systems, 1:300) and mouse anti-EB1 (clone 5, BD Bioscience, 1:1000) antibodies. Transfected RPE-1 cells (24h) were fixed with ice-cold methanol and incubated with rabbit anti-GFP (Roche, 1:10000) and mouse anti-EB1 (clone 5, BD Bioscience, 1:1000) antibodies. Cells were incubated with duolink PLA Probes anti-mouse PLUS and anti-rabbit MINUS (1:5) for 1h at 37°C and processed for ligation and rolling circle amplification (RCA) in the presence of cy5-labeled oligonucleotide probe according to manufacturer's protocol. Imaging was performed using a Zeiss Axiovert 200M fluorescence microscope with 100x oil objective and controlled using Metamorph 7.1.7 software. For quantification of RCP number, 5 different areas of at least 6 single cells from 3 independent experiments were analyzed.

### Statistical analysis

Statistical analyses were done using GraphPad Prism software. Data in bar graphs (mean +/− SD) and dot plots were analyzed using two-tail unpaired *t*-test. *p* < 0.05 was considered statistically significant.

## SUPPLEMENTARY MATERIAL TABLES, FIGURES AND MOVIES










