# Supplementary material for: Negative regulation of EB1 turnover at microtubule plus ends by interaction with microtubule-associated protein ATIP3
Source: Oncotarget. 2015 Oct 20;6(41):43557–70. doi: 10.18632/oncotarget.6196 (PMC4791250; doi:10.18632/oncotarget.6196)
Supplement: Supplementary file 1 [file oncotarget-06-43557-s001.pdf]

Negative regulation of EB1 turnover at microtubule plus ends by interaction with microtubule-associated protein ATIP3

Supplementary Material

Cell lines, plasmids and transfections

RPE-1 cells stably expressing EB1-GFP were a kind gift of Dr Matthieu Piel (Institut Curie, Paris, France). SV-MRC5, MCF7, HC7, HeLa and RPE-1 cells were described elsewhere [1]. All cells were used at passages 2 to 20 after thawing and grown as described [1]. Cells were routinely authenticated by morphologic observation and tested for absence of mycoplasma contamination using MycoAlert Assay detection kit (Lonza, France).

EB3-GFP and GFP-EB3 constructs (described in [2]) and expression vector encoding GST-EB1-ΔAc [3] were kind gifts of Dr Anna Akhmanova (Utrecht University, The Netherlands).

For rescue experiments of ATIP3 knock-down, HeLa cells were transfected with specific ATIP3-siRNA (sens strand UGG CAG AGG UUU AAG GUU A) that targets the 5' untranslated sequence of ATIP3 and allows expression of wild type ATIP3 coding sequence.

Live cell imaging

SV-MRC5 co-transfected for 24h with mCh-ATIP3 and EB3-GFP were imaged by spinning disc confocal microscopy on a Nikon Ti-E (Nikon) with perfect focus system (PFS, Nikon) equipped with a Plan APO VC 60x 1.40 N.A. oil objective, a Yokogawa motorized CSU-X1-A1 confocal head, a Photometrics Evolve 512 EMCCD camera (Roper Scientific) and controlled with MetaMorph 7.5 software (Molecular Devices). For simultaneous excitation of GFP and mCh we used 491nm 50mW Calypso (Cobolt) and 561nm 50mW Jive (Cobolt) lasers together with a DV2 beam splitter (MAG Biosystems, Roper) equipped with a dichroic filter 565dcxr (Chroma) and a HQ630/50m emission filter (Chroma). To keep cells at 37°C we used a stage top incubator (model INUG2E-ZILCS, Tokai Hit). Time lapse inset images were denoised using the ImageJ Safir Filter plugin [4]. Four iterations and a patch size of 1 were used as parameters of the denoising process.

For backtracking experiments, HC7 cells that stably express moderate levels of GFP-ATIP3 [1] were imaged on a Nikon Eclipse Ti-E with the PFS, equipped with a Nikon CFI Apo TIRF 100X 1.49 N.A. oil objective (Nikon), a TIRF-E motorized TIRF illuminator modified by Roper Scientific France/PICT-IBiSA (Institut Curie, Paris, France), a stage top incubator

(model INUG2E-ZILCS, Tokai Hit) set at 37°C, a Photometrics Evolve 512 EMCCD camera and controlled with MetaMorph 7.7 software. For excitation of GFP we used a Cobolt Calypso 491 nm (100 mW) laser and green fluorescent light was collected via a ET-GFP filter set (Chroma). Inset montages were denoised using the ImageJ Safir Filter plugin [3]. Four iterations and a patch size of 1 were used as parameters of the denoising process.

For analysis of EB1 decoration time, EB1 comet length and MT growth rate were measured from time-lapse TIRF videomicroscopy analysis (2 independent experiments) of HeLa cells co-transfected with EB1-GFP and control (siCtrl, n=10 cells) or ATIP3-specific (siATIP3, n=7 cells) siRNAs, as described previously [1].

Fluorescence recovery after photobleaching (FRAP) analyses

FRAP was performed on an inverted Ti-E (Nikon) Nikon spinning disk microscope. EB1 comets were bleached using a FRAP-on-fly-spiral option (Roper Scientific France/PICT-IBiSA), which allows bleaching of a selected circular region of 0.5 µm in diameter. We used 100% (7-15 kW/cm^2) of 491nm laser power for photobleaching. Time-lapse images were acquired for at least 3s before bleaching and for 10s after bleaching. Image acquisition was done at 150 ms of exposure.

For analysis, Safir software (Roper Scientific France) was used to denoise time-lapse images. Fluorescence recovery was measured in a region of 2 x 2 pixels that comprised part of the fluorescent MT end. Bleached MT ends and non-bleached cytoplasmic regions (background) were analyzed. To derive exponential decay curves for EB1-GFP, cytoplasmic background values were subtracted, and curves were normalized to 1. The average decay of fluorescence peaks was fitted as a first-order exponential decay with a robust fitting routine: $y = (y_0 - \text{plateau}) * \exp^{(-kdecay*x)} + \text{plateau}$, where y is normalized intensity, y_0 is the initial fluorescence value, plateau is the y value at infinite times, (y_0 – plateau) is the span of the reaction, k_{decay} is the reaction constant, and x is the time. Quantification was performed on bleached comets of 17 cells (siCtrl) and 16 cells (siATIP3) from 2 independent experiments.

The same experimental procedure and analysis were used to measure fluorescence recovery of cytosolic EB1-GFP signals, except that bleaching was performed in selected regions (2 x 2 pixels) of the cytosol.

Proximity Ligation Assay

In situ PLA detection was carried out using DUOLINK II In Situ Far Red kit (Sigma-Aldrich, St Louis, USA). RPE-1 cells stably expressing EB1-GFP were transfected for 24h with mCh or mCh-ATIP3, fixed in ice-cold methanol and incubated with rabbit anti-mCh (Clontech, 1:500) and/or mouse anti-GFP (Roche, 1:200) antibodies. Incubation with PLA probes and RCP analysis were performed as described in the Methods section.

Supplementary References

1. Molina A, et al. ATIP3, a novel prognostic marker of breast cancer patient survival, limits cancer cell migration and slows metastatic progression by regulating microtubule dynamics. Cancer Res. 2013; 73: 2905–2915.
2. Stepanova T, Slemmer J, Hoogenraad CC, Lansbergen G, Dortland B, De Zeeuw CI, Grosveld F, van Cappellen G, Akhmanova A, Galjart N. Visualization of microtubule growth in cultured neurons via the use of EB3-GFP (end-binding protein 3-green fluorescent protein). J. Neurosci. 2003; 23:2655–2664.
3. Komarova Y, Lansbergen G, Galjart N, Grosveld F, Borisy GG, Akhmanova A. EB1 and EB3 control CLIP dissociation from the ends of growing microtubules. Mol Biol Cell. 2005; 16: 5334–5345.
4. Kervrann C, Boulanger J. Optimal spatial adaptation for patch-based image denoising. IEEE Trans Image Process. 2006; 15: 2866–2878.

Supplementary Table S1. Characterization of ATIP3 domains and deletion mutants

Sequence	Positions (AA)	Length (AA)	EB1 binding	MT localization	Loss of EB1 comets
ATIP3	1-1270	1270	+	+	+
D2	410-874	465	+	+	+
D2N	410-634	225	-	+	nd
D2C	705-874	170	+	+	+
CN	705-816	112	+	+/-	+
CC	817-874	58	-	-	-
CN67	743-809	67	+	-	+
CN45	755-799	45	+	-	+
ATIP3delCN	[1-704]+[817-1270]	1158	-	+	nd
D2delCN	[410-704]+[817-874]	353	-	+	-
D2delCC1	[410-816]+[867-874]	413	+	nd	nd
delCC1	[1-816]+[869-1270]	1218	+	nd	nd
delCC2	[1-867]+[875-1270]	1263	+	nd	nd
delCTer	1-1240	1240	+	nd	nd
Del3P	[743-777]+[783-809]	61	-	-	-

AA: amino acids. Positions are according to accession number NP_001001924; nd: not determined.

Supplementary Table S2. *In vitro* microtubule dynamics in the presence of EB1-GFP and CN45 peptide

	Growth rate µm.min^{-1}	Shortening rate µm.min^{-1}	Catastrophe frequency events.min^{-1}
EB1-GFP 100 nM	2,15 ± 0,36 n=43	29,51 ± 9,78 n=38	0,282 n=39
EB1-GFP 100 nM + CN45 50nM	2,16 ± 0,34 n= 60	31,18 ± 9,81 n=55	0,254 n=60
EB1-GFP 100 nM + CN45 1µm	2,15 ± 0,38 n=65	33,81 ± 9,99 n=56	0,249 n=60

Growing and shortening rates were determined from kymograph slopes corresponding to individual growth or shrinkage phases (mean ± SD). Catastrophe frequencies were calculated as the number total of events divided by time spent in growth. n, number of events measured for each condition. Rescue events were not detected. The total time of measurements (growing and shrinkage phases) were: 149,14 min, 253,64 min and 255,81 min for EB1-GFP 100 nM, EB1-GFP 100 nM + CN45 50 nM and EB1-GFP 100 nM + CN45 1µM, respectively.

Supplementary Table S3: Oligonucleotides used for constructing ATIP3 domains and mutants

Name	Aim	Primers (5' → 3')	Number	Size (bp)
D2	PCR	F : CCGCTCGAGGA CTGACTTGGGATGCAAATGAT<br>R : CGGGGTACCTCATGCATTAAGAGCTGTAAATAA	C 206<br>C 208	1395
D2N	PCR	F : CCG CTCGAGGACTGACTTGGGATGCAAATGAT<br>R : CGGGGTACCTCAAAACAACGCAGAAACGGACCCGGT	C 206<br>C 304	675
D2C	PCR	F : CCGCTCGAGCAACCACCTCAGGTAGGAATATATCC<br>R : CGGGGTACCTCATGCATTAAGAGCTGTAAATAA	C 306<br>C 208	510
CN	PCR	F : CCGCTCGAGCAACCACCTCAGGTAGGAATATATCC<br>R : CGGCGGGGTACCTCAATTGTTGCTGTAAGTGCTCAGCTC	C 746<br>C 791	336
CC	PCR	F : GGCCCGCTCGAGGATCTGGTAATGCCGCTGTCATC<br>R : CGGGGTACCTCATGCATTAAGAGCTGTAAATAA	C 793<br>C 208	174
CN67	PCR	F : GCCCCGCTCGAGGAAGAAATCCCAGTGCTGATCGAGCC<br>R : CGGCGGGGTACCTCACTCACTGTGGGTGCTGGCTATTGA	C 987<br>C 988	201
CN45	PCR	F : GGCCCGCTCGAGGAAGGATCAGGCGTGTGTCCAG<br>R : CGGCGGGGTACCTCAGCTTCCTGTCCTCCGCAGCGC	C 1022<br>C 1023	135
Del3P	SDM	F : GCACAGTCGTCATGGGTGAATAAATCCAAAGCATCTTTG<br>R : CAAAGATGCTTTGGATTTATTCACCCATGACGACTGTGC	C 1030<br>C 1031	183
ATIP3delCN	SDM	F : GGGCTCTGCTTCAAAAACAACGTCTGGTAATGCCGCTGTC<br>R : GACAGCGGCATTACCAGACGTTGTTTTTGAAGCAGAGCCC	C 928<br>C 929	3474
ATIP3delCC1	SDM	F : AGCTGAGCACTTACAGCAACAATGGTCCTTCGAGA<br>R : TCTCGAAGGACCATTGTTGCTGTAAGTGCTCAGCT	C 724<br>C 725	3654
ATIP3delCC2	SDM	F : GATGAAAACTCCTCCAAAAGTTGAAAAGAGCAGGCAAAAG<br>R : CTTTTGCCTGCTCTTTTCAACTTTTGGAGGAGTTTTCATC	C 91<br>C 92	3789
ATIP3delCTer	SDM	F : CCGCTCGAGCCATGACTGATGATAATTCAGATG<br>R : CGGGGTACCTCAACACAGGTCCCCATTGTGCAG	C 70<br>C 209	3721
SANP	SDM	F : CCCACATCCTCCGCCAACCCTTTGCAGTCACCAAGGAATTCG<br>R : CGAATTCCTTGGTGACTGCAAAGGGTTGGCGGAGGATGTGGG	C660<br>C661	3813
SAIA	SDM	F : CCCACATCCTCCGCCATCGCTTTGCAGTCACCAAGGAATTCG<br>R : CGAATTCCTTGGTGACTGCAAAGCGATGGCGGAGGATGTGGG	C662<br>C663	3813
D2delCN	SDM	F : GGGCTCTGCTTCAAAAACAACGTCTGGTAATGCCGCTGTC<br>R : GACAGCGGCATTACCAGACGTTGTTTTTGAAGCAGAGCCC	C 928<br>C 929	1059
D2delCC1	SDM	F : AGCTGAGCACTTACAGCAACAATGGTCCTTCGAGA<br>R : TCTCGAAGGACCATTGTTGCTGTAAGTGCTCAGCT	C 724<br>C 725	1239

PCR : Polymerase Chain Reaction (subcloning) ; SDM : site-directed mutagenesis ; F : Forward ; R : Reverse

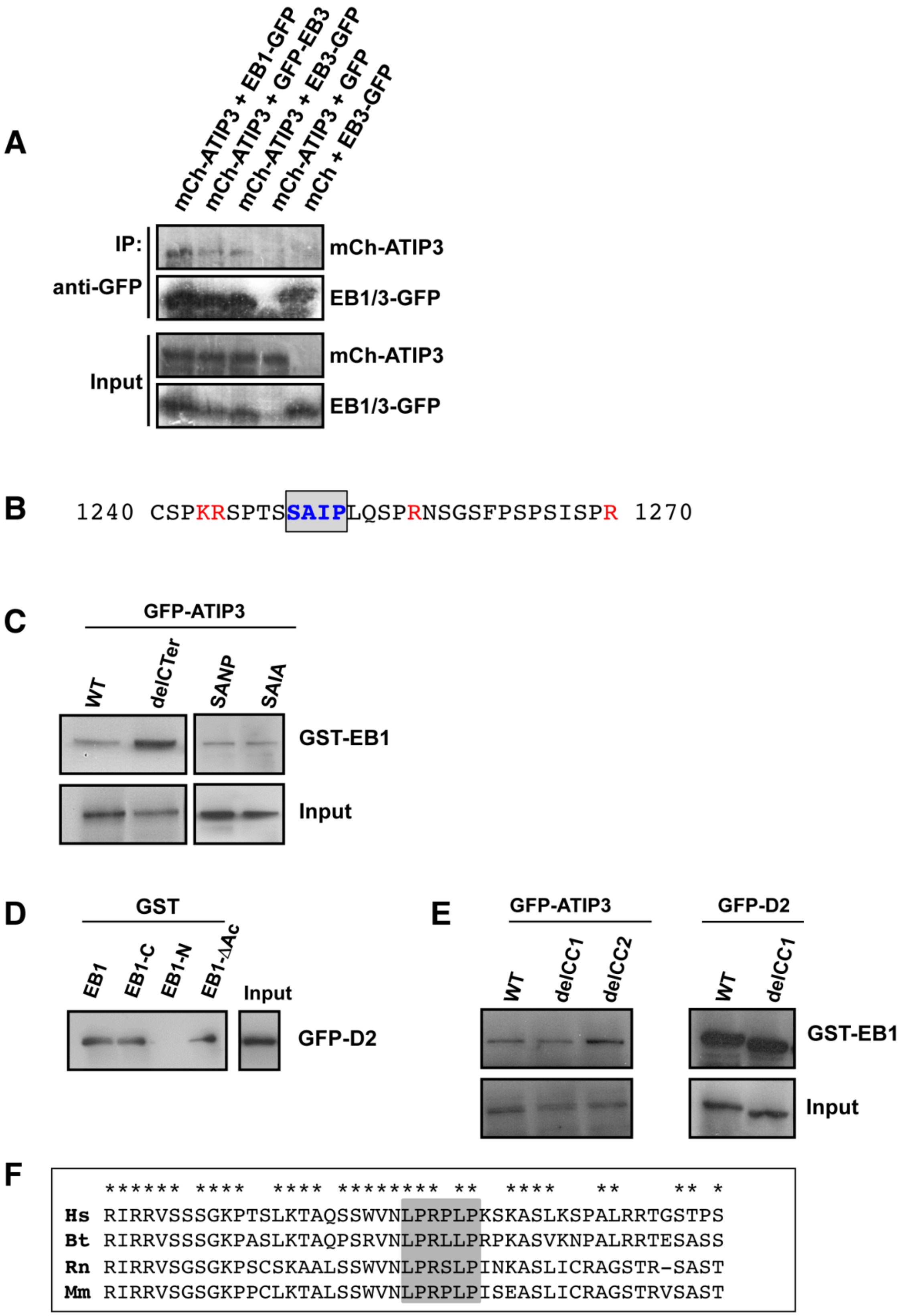

Supplementary Figure S1. ATIP3 interacts with EB1

(A). Co-immunoprecipitation of mCh-ATIP3 with EB1-GFP, EB3-GFP and GFP-EB3 using anti-GFP antibodies. MCF7 cells were transiently transfected with mCh-ATIP3 or mCh plasmids in the presence of EB1-GFP, GFP-EB3, EB3-GFP or GFP as indicated. Western blots were probed with anti-MTUS1 and anti-GFP antibodies to reveal mCh-ATIP3 (210 KDa) and EB1/3-GFP (55 KDa), respectively.

(B). Amino acid sequence of the C-Terminal portion of ATIP3. The SAIP motif (residues 1249-1252, in blue) is boxed. Basic residues are shown in red. Amino acid numbering is from Accession number NP_001001924.

(C). GST pull-down analysis showing precipitation of GFP-ATIP3 mutants using purified GST-EB1 agarose beads. Blots were probed with anti-GFP antibodies.

(D). GST pull-down assays of GFP-D2 expressed in MCF7 cells using purified GST-EB1 and deletants GST-EB1-C, GST-EB1-N and GST-EB1-ΔAc as indicated. Blots were probed with anti-GFP antibodies.

(E). GST pull-down analysis showing precipitation of GFP-ATIP3 and GFP-D2 mutants as indicated, using purified GST-EB1 agarose beads. Blots were probed with anti-GFP antibodies.

(E). Amino acid sequence alignment of the CN45 polypeptide with ATIP3-related sequences from different species. The proline-rich motif is boxed. Stars indicate conserved residues. Hs, Homo sapiens, Bt, Bos taurus, Rn, Rattus norvegicus, Mm, Mus musculus.

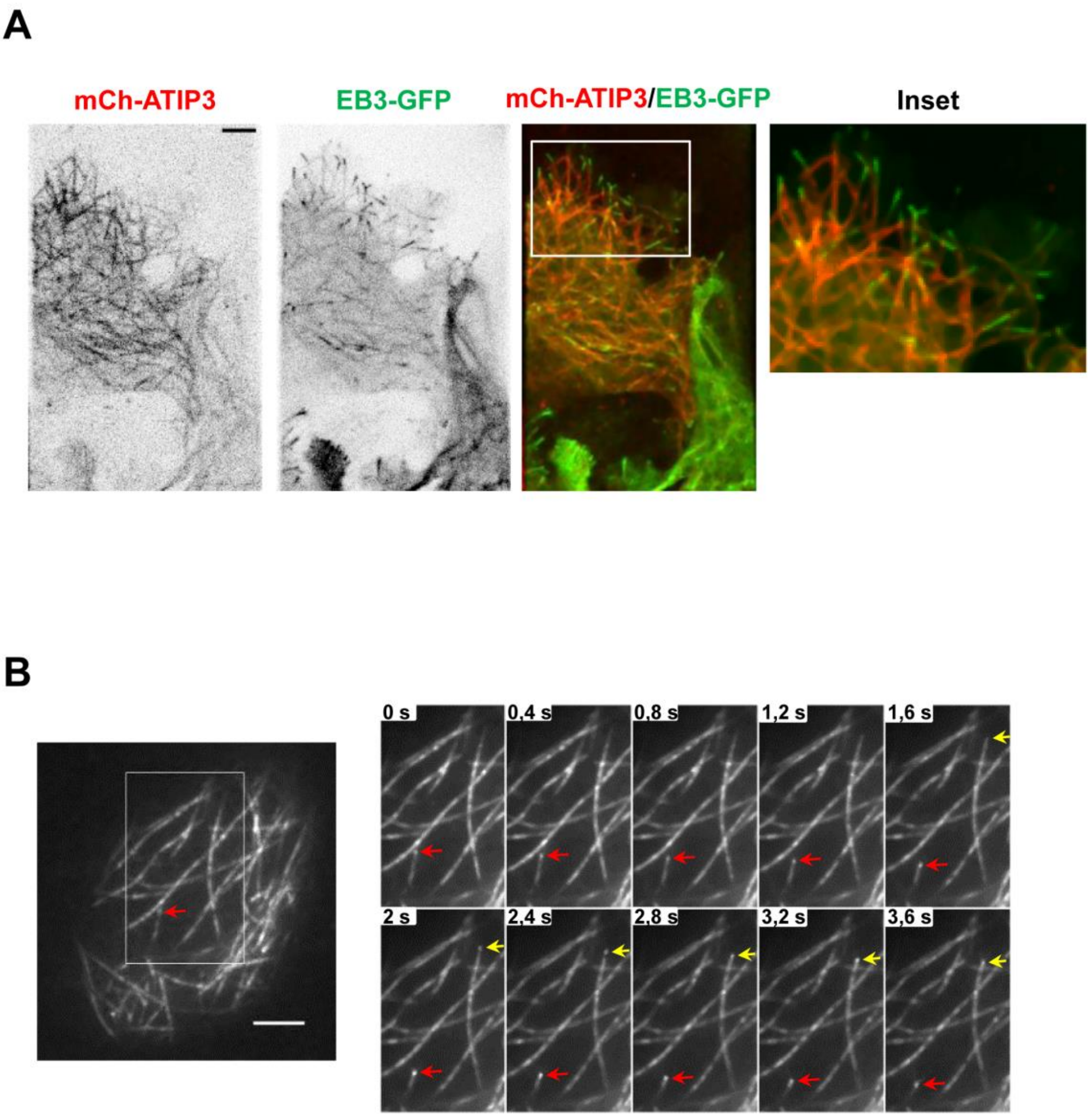

Supplementary Figure S2. ATIP3 is not a +TIP

(A). Images from fluorescent microscopy of SV-MRC5 living cells co-transfected with mCh-ATIP3 and EB3-GFP. Note that EB3 is enriched at the plus ends and weakly stains the MT lattice. Inset shows that mCh-ATIP3 is along the MT lattice and does not accumulate with EB3 at the plus ends.

(B). Time-lapse images of HC-7 cells (MCF7 stably expressing GFP-ATIP3 at endogenous levels). GFP-ATIP3 stains the MT lattice and the ends of shrinking MTs (arrows) in living cells, indicating backtracking properties. Scale bar, 10μm.

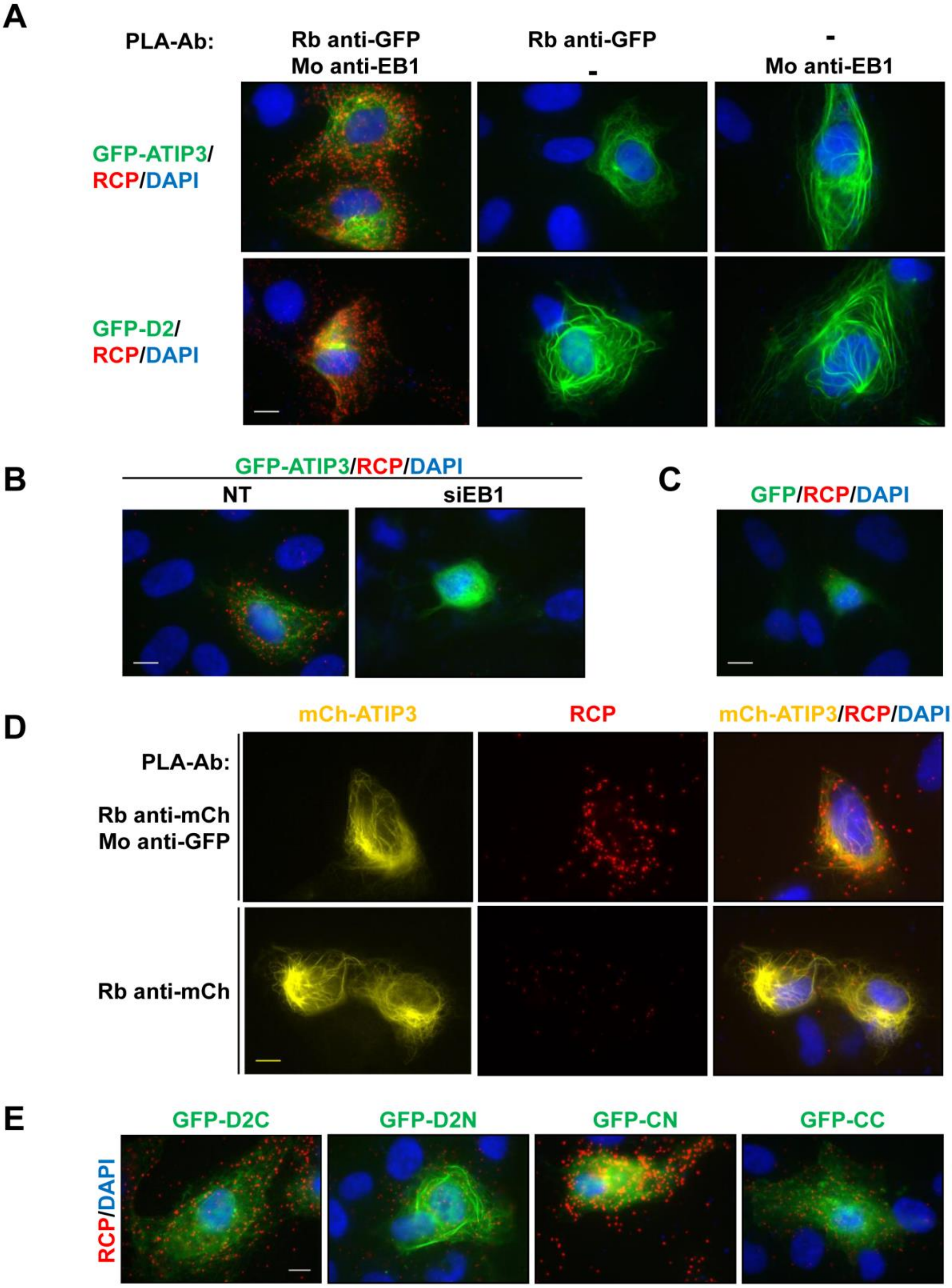

Supplementary Figure S3. ***In situ*** **ATIP3/EB1 molecular complexes**

(A). PLA performed in RPE-1 cells transfected with GFP-ATIP3 or GFP-D2 as indicated. Molecular complexes were analyzed using rabbit (Rb) anti-GFP and mouse (Mo) anti-EB1 primary antibodies as indicated, and corresponding secondary PLA antibodies. Rabbit anti-GFP or mouse

anti-EB1 primary antibodies alone were used as negative control. *In situ* molecular interaction is revealed by red bright signals of rolling circle amplification products (RCP) stained with cy5-labeled oligonucleotide probe. Shown are merge pictures of GFP (green), RCP (red) and nuclei (DAPI, blue) staining.

(B). PLA performed in RPE-1 cells transfected with GFP-ATIP3 in the presence or not of EB1-specific siRNA as indicated above. Shown are merge pictures as in (A).

(C). PLA performed in RPE-1 cells transfected with GFP and analyzed as in (A).

(D). PLA performed in RPE-1 cells stably expressing EB1-GFP and transfected with mCh-ATIP3. Molecular complexes were analyzed using rabbit (Rb) anti-mCh and mouse (Mo) anti-GFP primary antibodies and corresponding secondary PLA antibodies. Rabbit anti-mCh antibodies alone were used as negative control. *In situ* molecular interaction is revealed by red bright signals of rolling circle amplification products (RCP) stained with cy5-labeled oligonucleotide probe. Shown are merge pictures of mCh (yellow), RCP (red) and nuclei (DAPI, blue) staining.

(E). PLA performed in RPE-1 cells transfected with GFP-D2C, D2N, CN and CC constructs and analyzed as in (A). Shown are merge pictures as in (A).

(A-E). Magnification x100. Scale bar 10 µm.

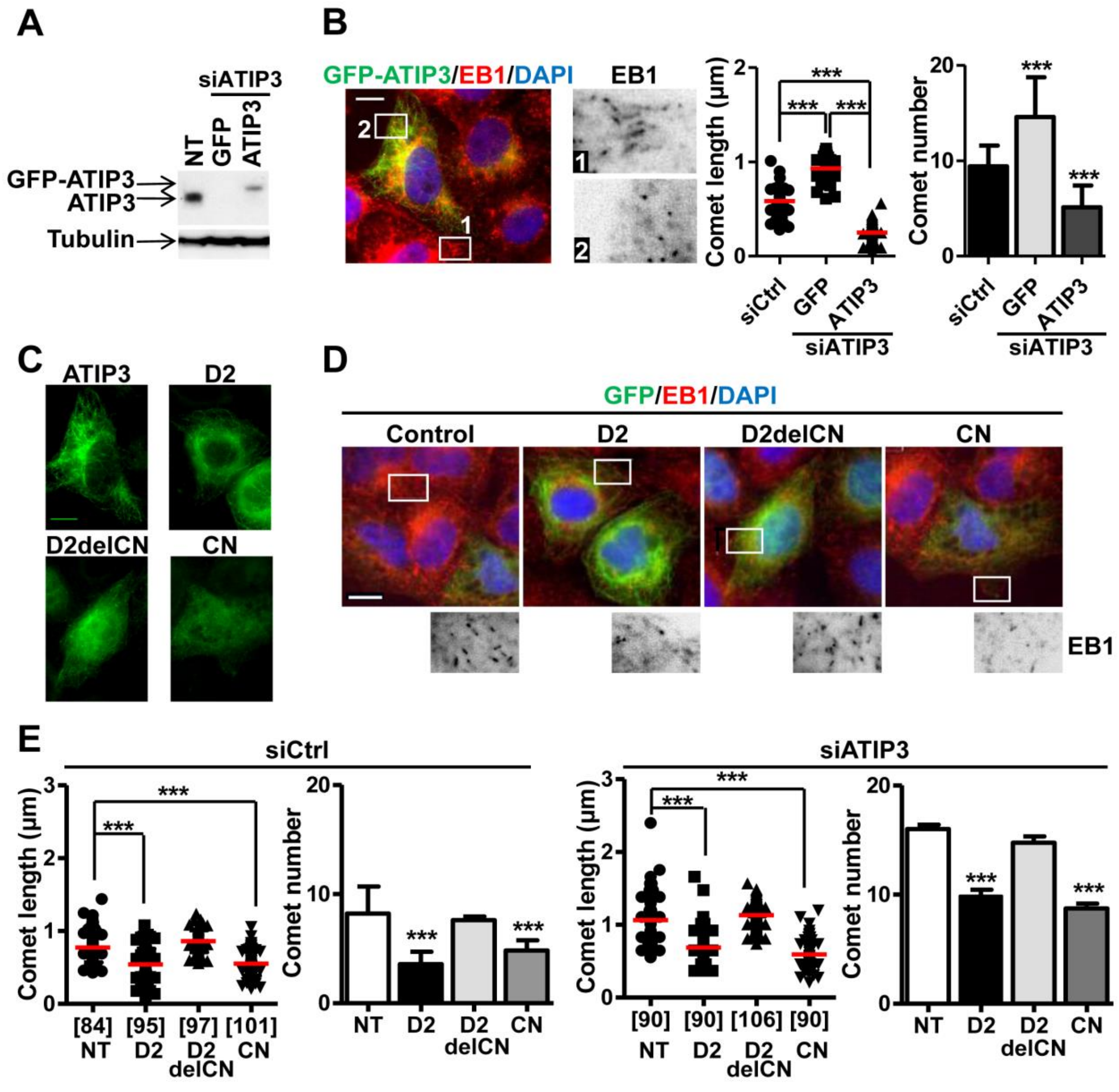

Supplementary Figure S4. Rescue of ATIP3-silenced phenotype

(A). Western blot analysis comparing endogenous ATIP3 and GFP-ATIP3 expression levels. HeLa cells were either left non-transfected (NT), or transfected for 72h with ATIP3-specific siRNA (siATIP3) and for 24h with plasmids encoding GFP (0.5 µg) or GFP-ATIP3 (2 µg) as indicated. Blots were probed with anti-MTUS1 antibodies to reveal endogenous ATIP3 (180 KDa) and transfected GFP-ATIP3 (210 KDa), and then reprobed with anti-tubulin antibodies for internal control.

(B). ATIP3-silenced HeLa cells were transfected with GFP-ATIP3 plasmid (2 µg) as in (A), and staining with anti-GFP (green), anti-EB1 (red) and DAPI (blue) was performed. Insets show EB1 comet-like structures in enlarged portions of selected areas (insets 1 and 2 show non-transfected and transfected cells, respectively). Right panel: Quantification of comet length (scattered dot plot) and number of comets (per 62µm2 area). For each condition, fifty comets were analyzed from 5 to 6 single cells, in 3 independent experiments. *** p<0.0001.

(C). ATIP3-silenced HeLa cells were transfected with GFP-ATIP3 (2 µg), GFP-D2 (2 µg), GFP-D2delCN (1µg) or GFP-CN (0.5 µg) as indicated. Fluorescence intensity of GFP immunostaining indicates similar levels of expression for all fusion proteins.

(D). ATIP3-silenced HeLa cells were transfected for 24h with indicated domains in conditions of moderate expression as shown in (C). Staining with anti-GFP (green), anti-EB1 (red) and DAPI (blue) was performed. Insets (below) show EB1 accumulation at the MT plus-end in enlarged portions of selected areas.

(E). Quantification of comet length (scattered dot plot) and number of comets (per 62μm^2 area) in HeLa cells transfected with control siRNA (siCtrl) (left) or ATIP3-specific siRNA (siATIP3) (right panel) then transfected with GFP fusion proteins at levels close to endogenous as in (C). Quantification is from 5 to 6 single cells in 3 independent experiments. Number of comets analyzed is under brackets. *** p<0.0001.

(B-D). Scale bar, 10 µm.

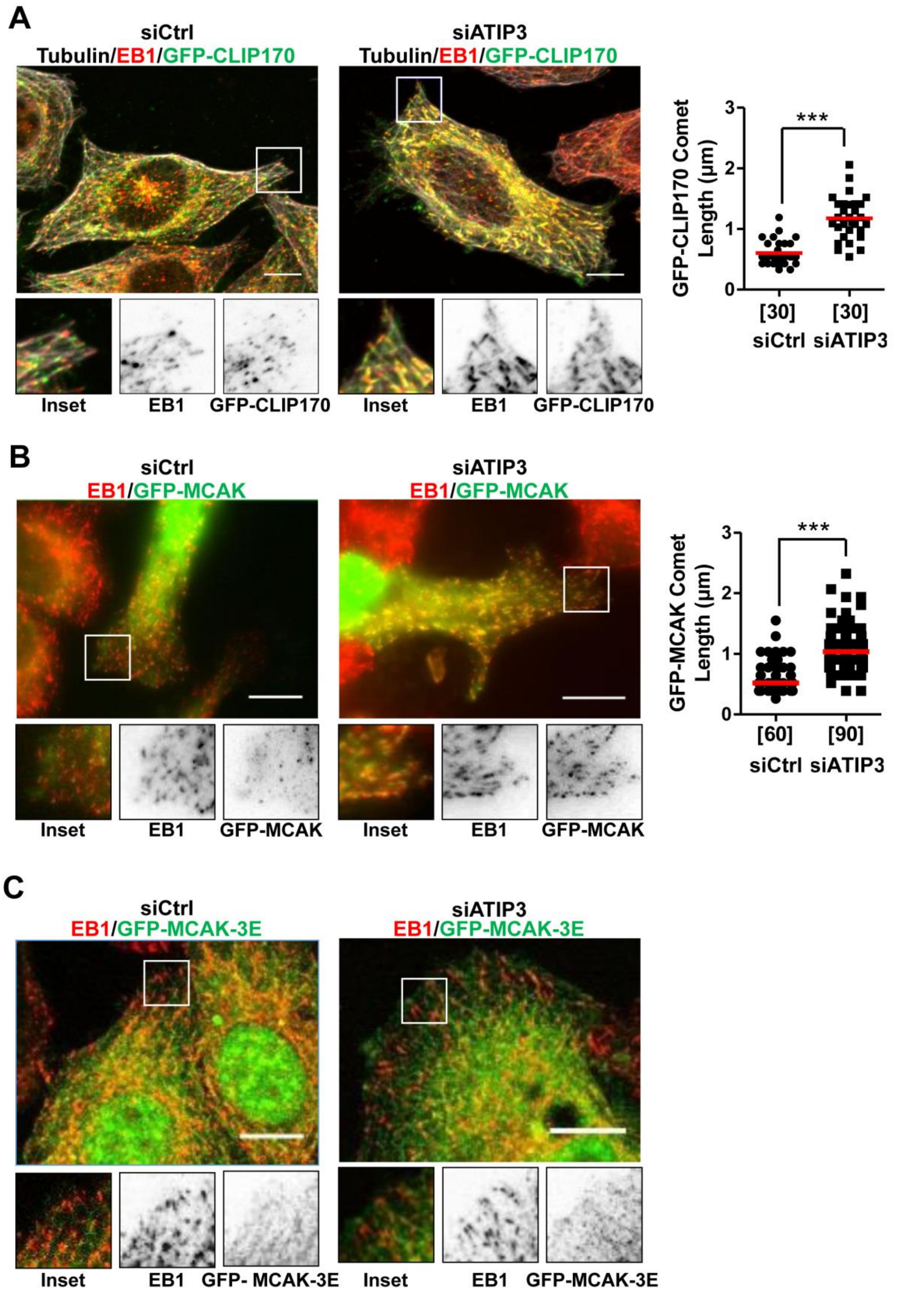

Supplementary Figure S5. ATIP3 silencing increases accumulation of CLIP-170 and MCAK together with EB1 at growing MT ends

(A). HeLa cells were transfected for 72h with control (siCtrl) or ATIP3-specific siRNA (siATIP3) and for 24h with GFP-CLIP-170 construct, then fixed and stained with anti-GFP (green), anti-EB1 (red) and anti-alpha-tubulin (grey). Insets show GFP-CLIP170 and EB1 comet-like structures in enlarged portions of selected areas. Scale bar: 10 µm. Quantification of GFP-CLIP-170 comets in control and ATIP3-silenced cells is shown on the right. Comets were analyzed from 5 single cells in 2 independent experiments. Number of comets analyzed is under brackets. ***p<0.0001 .

(B). HeLa cells were transfected for 72h with control (siCtrl) or ATIP3-specific siRNA (siATIP3) and for 24h with GFP-MCAK construct, then fixed and stained with anti-GFP (green) and anti-EB1 (red). Insets show GFP-MCAK and EB1 comet-like structures in enlarged portions of selected areas. Scale bar: 10 µm. Right panel Quantification of GFP-MCAK comets as in (A). ***p<0.0001.

(C). HeLa cells were transfected for 72h with control (siCtrl) or ATIP3-specific siRNA (siATIP3) and for 24h with construct encoding mutant MCAK-3E that does not bind EB1, then processed as in (B).

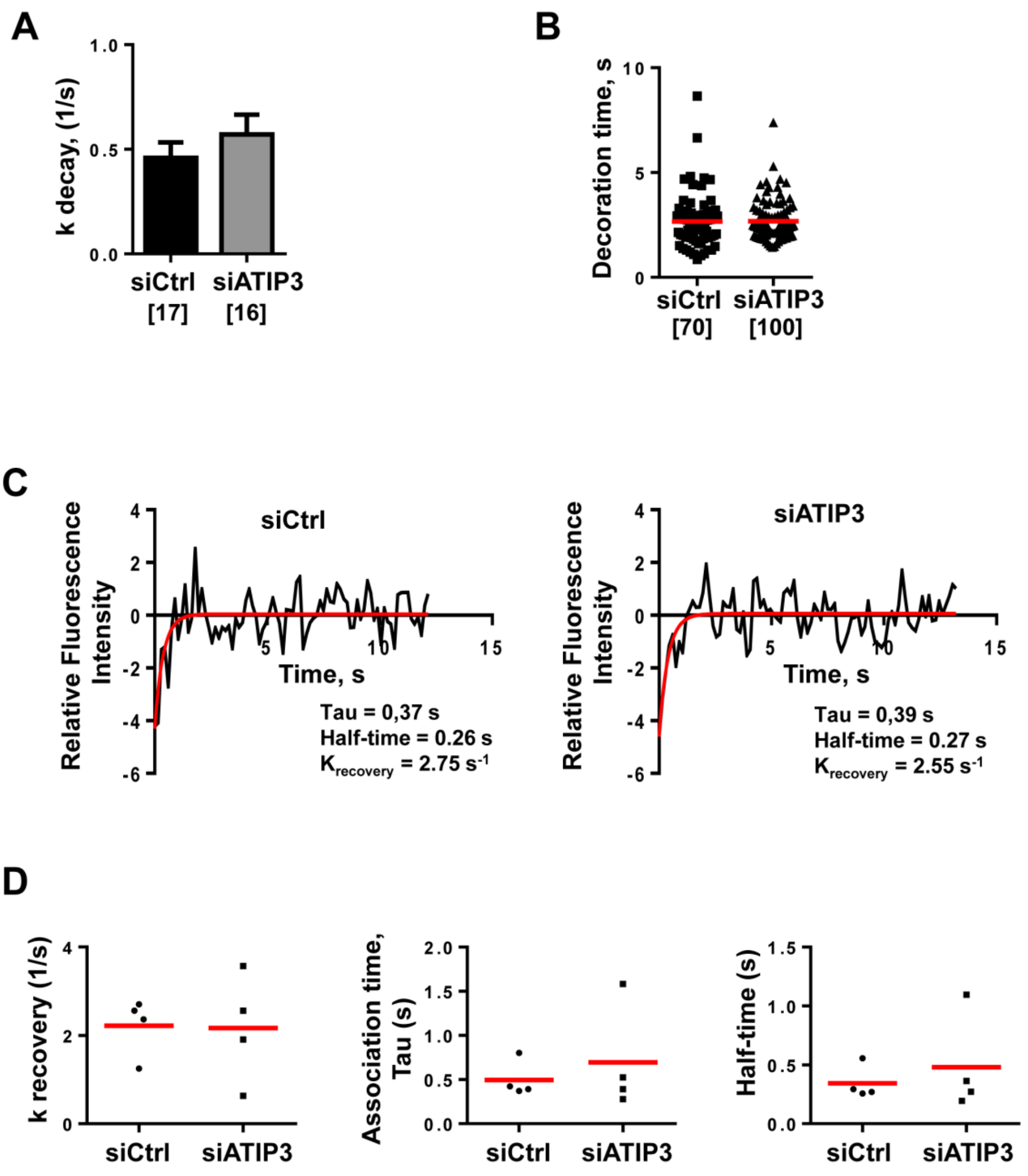

Supplementary Figure S6. EB1-GFP fluorescence decay, decoration time and EB1-GFP fluorescence recovery in the cytosol are not modified upon ATIP3 depletion

(A). Quantification of k_{decay} (s^{-1}) of EB1-GFP fluorescence decay measured by FRAP analysis in control (siCtrl, n=17) and ATIP3-depleted (siATIP3, n=16) cells, from 2 independent experiments. Number of EB1-GFP comets analyzed is under brackets.

(B). Quantification of EB1 decoration time (s) calculated as the ratio of comet length to microtubule growth rate. Time-lapse TIRF videomicroscopy analysis of HeLa cells co-transfected with siRNAs (control (siCtrl) or ATIP3-specific (siATIP3)) and EB1-GFP is described in Molina et

al. [17]. Quantification was performed on 10 cells (siCtrl) and 7 cells (siATIP3) from 2 independent experiments. Number of EB1-GFP comets analyzed is under brackets.

(C). Representative fluorescence recovery curves (in red) after photobleaching EB1-GFP in selected areas of the cytosol of control (siCtrl) and ATIP3-depleted (siATIP3) HeLa cells.

(D). Quantification of $k_{recovery}$ (s^{-1}), association time Tau in seconds (s) and half-time of recovery in seconds (s), of EB1-GFP fluorescence recovery in the cytosol of control and ATIP3-depleted cells. For each condition, 4 bleached areas were analyzed from 4 single cells in 2 independent experiments.

Legends to Supplementary Movies

Supplementary Movie 1. Time-lapse microscopy of SV-MRC5 cells co-transfected with mCh-ATIP3 (red) and EB3-GFP (green).

EB3-GFP (left) and mCh-ATIP3 (right) MRC5-SV-coexpressing cells were imaged by spinning disk confocal microscopy (60X lens). Time-lapse series of 100 images were acquired in a stream mode and shown at 15 frames per second.

Supplementary Movie 2. Time-lapse microscopy of SV-MRC5 cells co-transfected with mCh-ATIP3 (red) and EB3-GFP (green). Inset.

mCh-ATIP3 (red) and EB3-GFP (green) MRC5-SV-coexpressing cells were imaged by spinning disk confocal microscopy as for Supplementary Movie 1. A denoised zone of 1900 µm2 at the cell periphery is shown.

Supplementary Movie 3. Time-lapse microscopy of HC-7 cells stably expressing moderate levels of GFP-ATIP3.

Stably transfected GFP-ATIP3 cells were analyzed by time-lapse TIRF microscopy (100X lens). Time-lapse series of 500 images were acquired in a stream mode and shown at 30 frames per second.

Supplementary Movie 4. Time-lapse videomicroscopy of EB1-GFP fluorescent recovery after photobleaching in control and siRNA-silenced HeLa cells.

HeLa cells were co-transfected with EB1-GFP and siRNA (control (siCtrl) or ATIP3-specific (siATIP3)) for 24h and 72h, respectively. EB1-GFP fluorescence recovery following photobleaching was analyzed by Spinning disk microscopy (100X lens). Red arrowhead indicates bleached comet. Time-lapse series of 105 images were acquired with a 150 ms interval and shown at 2 frames per second.
